# Binge-Watching: Development and Validation of the Binge-Watching Addiction Questionnaire

**DOI:** 10.3390/bs11020027

**Published:** 2021-02-23

**Authors:** Giuseppe Forte, Francesca Favieri, Domenico Tedeschi, Maria Casagrande

**Affiliations:** 1 Dipartimento di Psicologia, Università “Sapienza” di Roma, 00185 Roma, Italy; francesca.favieri@uniroma1.it (F.F.); angelious@hotmail.it (D.T.); 2 Dipartimento di Psicologia Dinamica, Clinica e Salute, Università “Sapienza” di Roma, 00185 Roma, Italy

**Keywords:** binge-watching, binge-watching addiction, addictive behavior, addiction, tv series, questionnaire validation, factor analysis

## Abstract

The approach to the vision of TV series has deeply changed in the last years, and watching multiple episodes of TV content in a single session becomes a popular viewing pattern referred as binge-watching. Early studies defined binge-watching as a potentially addictive behavior showing characteristics similar to other behavioral addictions, such as loss of control and pleasure anticipation. This study aims to validate a short self-report questionnaire focused on assessing binge-watching behavior and determining whether it shows characteristics similar to addictive behavior, the Binge-Watching Addiction Questionnaire (BWAQ). An online survey was adopted to administer the questionnaire in the general population (N = 1277). Exploratory and confirmatory factor analyses assessed both the validity and the structure of the scale in two independent samples. The statistical analyses confirmed a four-factor model (i.e., “Craving”, “Dependency”, “Anticipation”, “Avoidance”) of the BWAQ with good psychometric properties. The BWAQ can differentiate between people who adopt maladaptive watching activities from those who use TV-series as leisure and entertainment activities. Therefore, this questionnaire may enable researchers to improve this emerging field of research significantly.

## 1. Introduction

In the last few decades, a broad diffusion of new technological devices and on-demand services for entertainment deeply changed the TV content approach. A wide variety of TV screenplays permanently available, and the intense and consecutive viewing of several episodes of one TV series, has become a popular viewing pattern in the on-demand platforms (e.g., Netflix, PrimeVideo, Hulu) [1,2,3]. This behavioral phenomenon is defined as binge-watching (BW). Although no agreement exists about the definition of BW, several authors suggested that the principal criterion for identifying it is to watch in one sitting three or more episodes of the same TV series or content [4,5,6].

Despite the term “binge” usually referring to negative aspects of excessive and harmful behavior (e.g., binge eating, binge drinking), the BW would represent a typical consumption pattern of video content in current society [7]. Accordingly, between 63 and 73 percent of American citizen and about 51 percent of European citizens declare to adopt BW behaviors [8,9,10], and about 60 percent of binge-watchers, especially young adults and college students, report this behavior more than once a week [11,12,13,14].

Many authors tried to highlight the main BW characteristics, considering causes, effects, environmental and motivational factors, as well as the impact of the pattern on psychological well-being [1,6,10,12,14,15,16,17,18,19,20]. Some studies, attempting to define the risks and benefits of BW, focused on its negative aspects. Accordingly, they underlined the potential role of this behavioral pattern as a risk factor for health and daily functioning (e.g., [2,21,22]). Sedentary lifestyles, maladaptive eating behaviors, and some adverse effects on psychological and social well-being appear to be associated with BW, as confirmed by the higher levels of anxiety, depression, and social isolation reported in binge-watchers than in the general population (e.g., [23,24]). Although higher satisfaction in individuals who reported BW behavior was highlighted [25], some authors consider binge-watching a potentially addictive behavior [26,27]. Although some BW aspects are considered clinically relevant, some characteristics do not fully meet the behavioral addiction criteria.

For this reason, some authors place the behavior under attention for its possible declination as addiction [26,27]. Griffiths [28], considering a higher number of potentially addictive behaviors, postulated that behavioral addiction consisted of some common components, such as salience, mood modification, tolerance, withdrawal, conflict. No evidence confirmed the presence of these components in the BW. However, previous studies reported some characteristics ascribed to behavioral addiction (e.g., interpersonal problems, low self-control, social isolation, and the alteration in daily activity [29,30,31]).

In line with the recent interest for this topic, specific tools to assess the BW were developed, i.e., (1) the Problematic Series Watching Scale (PSWS) [32], evaluating problematic TV series watching, (2) the Series Watching Engagement Scale (SWES) [33], measuring engagement in TV series vision, (3) the Watching TV Series Motives Questionnaire (WTSMQ), and (4) the Binge-Watching Engagement and Symptoms Questionnaire (BWESQ) [26], assessing TV series watching reasons and binge-watching engagement and symptoms. However, these previous assessment tools showed some limits. For example, the PSWS was based on the principal criteria of addiction (from DSM-5) referred to BW, while the SWES and WTSMQ appear more focused on motivational features or associated booster. Finally, although BWESQ seems to be an interesting tool to analyze BW traits and symptoms, and it is similar to the one we proposed, the presence of a global score that measures addiction behaviors is not reported. 

To overcome these limitations, this study proposes a new and short self-report questionnaire to assess binge-watching behavior and determine whether it shows the main characteristics typical of addictive behaviors. The Binge-Watching Addiction Questionnaire (BWAQ) was developed, and its factorial structure was evaluated in an Italian sample, adopting a factorial confirmatory approach.

## 2. Method

### 2.1. Participants

A web-based cross-sectional survey implemented using Google Form and broadcasted through mainstream social media (such as Facebook, Twitter, Instagram) was used to collect data among the Italian-speaking population. The survey was carried out from December 2018 to December 2019. Participants could withdraw from the study at any time without providing any justification, and no data was saved. Only the completed surveys were considered for the analysis. About 80 percent of the total respondents (1277/1859) who started the questionnaire completed the entire survey and were considered for statistical analyses. Due to the current exploratory aim, being at least 18 years old and being Italian were the only inclusion criteria. 

Finally, 1277 respondents participated in the study (mean age = 22.61; SD = 5.94; women = 73%). The original sample was split, based on age and gender, for cross-validation conducting an exploratory factor analysis (EFA) (mean age = 22.56; SD = 5.81; women = 73%) and a subsequent confirmatory factor analysis (CFA) (mean age = 22.67; SD = 6.07; women = 73%). Participants’ characteristics of the general sample, and each subsample are reported in Table 1.

### 2.2. Measures

#### 2.2.1. Demographic Questionnaire

The demographic questionnaire collected information about age, gender, years of education, and occupational and marital status to describe the sample.

#### 2.2.2. Binge-Watching Addiction Questionnaire (BWAQ)

A total of fifty initial items were created. The screening of behavioral addiction and impulse control disorders inspired their choice. In line with the current theoretical models of addictive behaviors, all the items were developed considering the main dimensions of the construct (such as craving, avoidance, anticipation, dependency, loss of control, mood alteration).

Then, to improve the validity and according to previous studies (e.g., [26]), a focus group, including regular TV series viewers (N = 10) and two clinical psychologists with expertise on addictive behavior, was conducted to identify the main aspects of this behavior. The focus group’s work allows defining the conceptualization and operationalization of items. Formally, items were based on the existing scale on Internet Addiction [34]. This process resulted in a 24-item, 5-point Likert scale, from 0 (never) to 4 (always), included for the factor analyses. At the bottom of the paper, the final version of the BWAQ in both English and Italian was reported (Table A1). 

#### 2.2.3. Other Questionnaires

The Barratt Impulsiveness Scale (BIS-11; [35]) was adopted to assess the personality/behavioral construct of Impulsiveness. Moreover, Beck Depression Inventory (BDI; [36]) was adopted to measure depression levels. Both impulsivity and depression are strongly related to addictive behaviors. 

### 2.3. Procedure

After the short demographic questionnaire, participants completed the BWAQ. All respondents were informed about the aims of the study and had to confirm their consent before starting the survey. No personal information, which could allow the identification of participants, was collected to guarantee anonymity.

All procedure was approved by the ethical committee of the Department of Dynamic and Clinical Psychology (“Sapienza” University of Rome; protocol number: 0000801) and was conformed to the Helsinki Declaration.

### 2.4. Data Analyses

Descriptive statistics of sociodemographic characteristics were computed (frequencies, mean and standard deviation of the variables). 

An analysis was conducted to reduce items and ensure the inclusion in the scale only of functional and internally consistent items. The deletion of items was supported by the estimation of inter-item and inter-total correlations. For these analyses, SPSS software was used.

The factorial structure of the scale was examined by exploratory (EFA) and confirmatory factor analyses (CFA). These analyses have been conducted in two subsamples. The EFA was computed in half of the sample, and the factors were derived from a principal component analysis and oblique rotation (Oblimin). Oblimin rotation was used because there was no reason to assume that the extracted factors were orthogonal. 

The scree plot obtained by the principal component analysis was used to determine the number of extracted factors. However, each factor with an eigenvalue equal to or higher than 1 was considered.

The number of factors suggested by the EFA was then cross validated in the CFA.

The maximum likelihood (ML) estimation was employed in CFA. Goodness-of-fit was assessed using chi-square, Comparative Fit Index (CFI), Tucker Lewis Index (TLI), and Standardized Root Mean Square Residual (SRMR), Root Mean Square Error of Approximation (RMSEA) indices [37]. Other normed fit indices included were: Incremental Fit Index (IFI), Goodness of Fit (GFI), Normed Fit Index (NFI). The cut-off criteria for the fit indices were based on Kline’s suggestions [37]. Cronbach’s alpha examined internal consistency Composite Reliability (CR), and Average Variance Extracted (AVE) of the identified factors were also calculated [38,39].

Pearson’s r correlations were calculated to describe the relationship between some sample’s characteristics (age, years of education) and the BWAQ global score. Moreover, the convergent validity of the BW construct was evaluated through the correlations with BIS-11 and BDI scores.

IBM SPSS Statistics (version 24.0) [40] and open-source software R [41] were used to perform statistical analyses in the current study.

## 3. Results

### 3.1. Descriptive Statistics and Item Reduction Analysis

Table 1 showed the descriptive statistics of the sample.

Cronbach’s alpha, calculated on the EFA sample, showed a good internal consistency (Cronbach’s α = 0.92). However, we removed 2 of the 24 items (i.e., (item 16) “*Have you tried to reduce the amount of time spent watching TV series?*”; (item 24) “*Can you stop watching your favorite TV series even when something important is going to happen in the plot?*”) because their removal improved the internal consistency (Cronbach’s α = 0.94). 

### 3.2. Exploratory Factor Analysis

An EFA was conducted initially on the 24-item version of BWAQ, but the following examination of each item confirmed the exclusion of the two items identified by the item reduction analysis because of their low factor loading (less than 0.30 [42]). Then, the final analysis was conducted on the 22-item version.

The Kaiser–Meyer–Olkin measure verified the sampling adequacy for the analysis (KMO = 0.934), and Bartlett’s test of sphericity indicated sufficiently large correlations between items (χ2 (276) = 7169.73, *p* < 0.0001).

**Figure 1:**
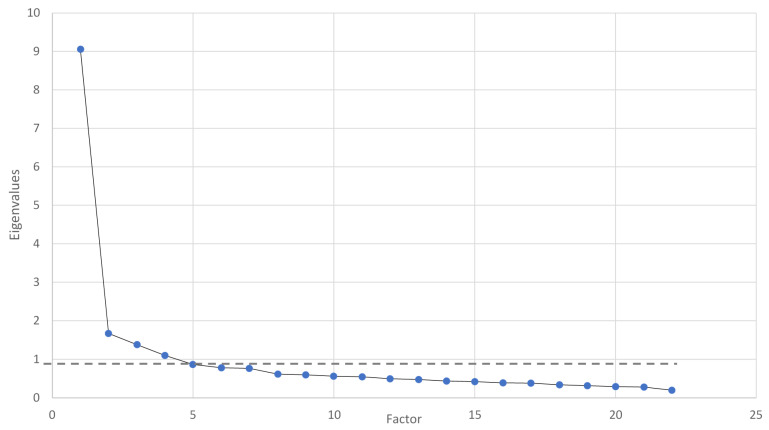
Scree Plot of EFA.

A first analysis was run to obtain eigenvalues for each component (Table 2). A total of four components had eigenvalues higher than 1 and explained 60.07 percent of the variance. The scree plot (Figure 1) was ambiguous and showed inflections that would justify retaining two and four factors. According to the convergence between the scree plot and eigenvalues, four components were considered in the final analysis.

Table 3 shows the factor loading of the items for each factor. 

### 3.3. Confirmatory Factor Analysis

A preliminary CFA was conducted according to the EFA results. However, the indices reported non optimal fit of the model according to thresholds (χ2/df = 6.08; CFI = 0.87; TLI = 0.85; RMSEA = 0.89 (CI 90% = 0.083–0.093); SRMR = 0.06; NFI = 0.86; IFI = 0.87; GFI = 0.84). For this reason, another CFA was calculated after deleting item 6 (“*Are your studies, your work, or your activities negatively affected by the amount of time you spend watching TV series?*”) because it saturated in two factors, and item 3 (“*Do you happen to prefer watching a TV series to relationships with your partner, friends, etc.?*”) because it was similar to the item 19. Lower AIC (Akaike information criterion) and BIC (Bayesian information criterion) indices (AIC and BIC of the first CFA: 39,345.35 and 39,559.42, respectively; AIC and BIC of the second CFA: 35,867.06 and 36,063.29, respectively) confirmed an improvement of the CFA model. 

The CFA fit indices of this analysis confirmed an optimal model fit (χ2/df = 4.98; CFI = 0.91; TLI = 0.89; RMSEA = 0.069 (CI 90% = 0.064–0.074); SRMR = 0.057; NFI = 0.88; IFI = 0.91; GFI = 0.87). In particular, SRMR (threshold acceptable fit < 0.08) and RMSEA (acceptable fit thresholds: between 0.05 and 0.08) reported a good fit value. As well as CFI, TLI, NFI. IFI and GFI showed a value near or beyond the threshold of 0.09, indicating that the texted model is acceptable in terms of these indices. The CR values were higher than the threshold of 0.75 (Factor 1 = 0.91; Factor 2 = 0.82; Factor 3 = 0.75; Factor 4 = 0.82), suggesting good construct reliability. The AVE values are equal or beyond the threshold of 0.50 ((Factor 1 = 0.53; Factor 2 = 0.53; Factor 3 = 0.50; Factor 4 = 0.53), indicating a good convergent validity. For these reasons, the proposed model met the criteria of feasibility and convergent validity and, overall, it successfully passed.

The CFA results cross-validated the four-factor structure derived from the EFA. Factor loadings were high for all the items and significant at an alpha level of 0.05 (see Table 4).

**Figure 2:**
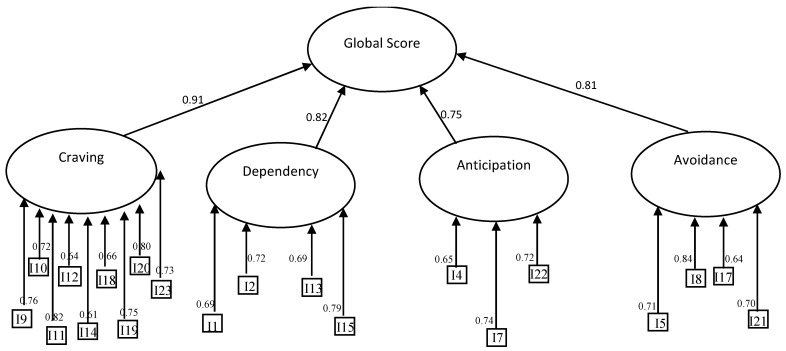
Second-order model for the BWAQ. The figure shows the four-factor model and the second-order factor in the confirmatory factor analysis. Rectangles indicate observed variables (items); ovals represent factors and the second-order model. All factors loading (standardized) are reported and are statistically significant (*p* < 0.01). Error terms are not presented for a simpler presentation.

The four-factor of the BWAQ had high internal consistency (Cronbach’s α were: Factor 1 = 0.91; Factor 2 = 0.82; Factor 3 = 0.75; Factor 4 = 0.81). The inter-factor correlation matrix showed significant positive correlations (see Table 5). This result could suggest a second-order overall factor of binge-watching. Accordingly, a second-order factor structure was tested (Figure 2). Since the first- and second-order models were substantially identical, the second-order model was accepted (according to [43]).

### 3.4. Interpretation of the Factors

We proceeded to examine the content of the 20 BWAQ items. According to this analysis, the four factors were *Craving*, *Dependency*, *Anticipation*, and *Avoidance* (see Table 4).

The *Craving* scale captures the degree of pleasure and mood during binge-watching. This scale considered the craving construct and, included the assessment of the intense desire to act and interpersonal problems consequent to binge-watching (e.g., “*How many times do you find yourself diverting your attention from negative thoughts with the consoling thought of your favorite TV series?”; “do you often feel depressed, irritable or nervous when you can’t watch a TV series?*”).

The *Dependency* scale refers to compulsive binge-watching and failure to control the behavior. It also covered household, sleep, and occupational-related problems (e.g., “*Do you happen to find yourself saying “just one more episode and I’ll turn it off” when you watch a TV series?*”; “*Do you happen to neglect household chores to spend more time watching TV series?*”). 

The *Anticipation* scale describes the search for cues related to the contents of the TV-series, even when the behavior is not implemented (e.g., “*Are you interested in new releases TV series?*”; “*Do you often read reviews and opinions about new TV series?*”). 

The *Avoidance* scale regards the lack of awareness about the problematic behavior and the tendency to minimize it (e.g., “*Do you happen to think that people overestimate the time you spend watching TV series?*”; “*Do you try to minimize or hide how much time you spend watching TV series?*”). 

The item scores of the final version of the BWAQ converge in a global index, representing the overall binge-watching addiction level. The sum of the item scores of each factor reflected the severity of addictive behavior in the corresponding dimension. 

### 3.5. Distribution of the Scores

**Figure 3:**
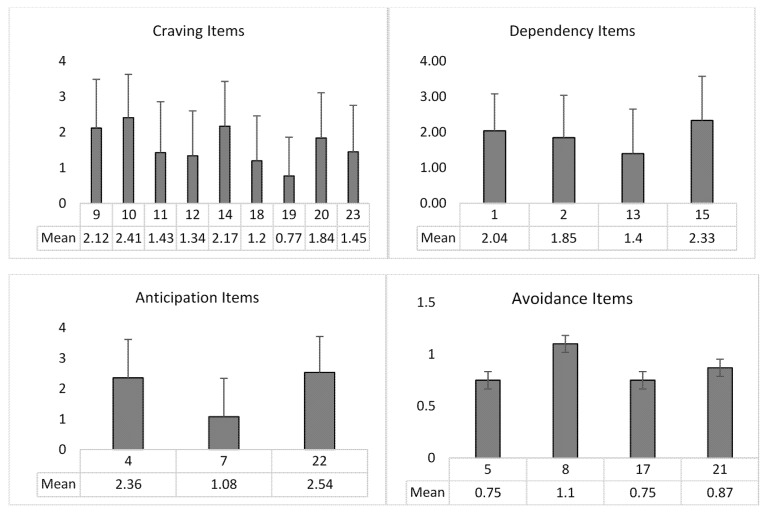
Mean and Standard Deviation of the single item scores for each factor.

The whole sample, consisting of the total respondents, was considered to define the distribution of the scores. Mean and standard deviation were computed for global score (33.42 ± 17.64) and the four subscales (Craving: 16.31 ± 10.29; Dependency: 7.64 ± 3.71; Anticipation: 5.99 ± 3.01; Avoidance: 3.48 ± 3.56) (see Table 6). Figure 3 showed the mean and standard deviation of the score reported for each item included in the factors. To define moderate or problematic BW behavior, a cut-off was calculated considering one or two standard deviations from the mean. A value higher than one and two standard deviations from the mean were considered over the cut-off point for moderately (global score: ≥51 and <69) and problematic (global score: ≥69) behavior, respectively (Table 6).

Adopting this cut-off point, a percentage of 14% (174/1277) of the sample presented moderately BW behavior, and 4% (45/1277) reported problematic levels of BW as a possible index of addictive behavior.

#### Correlational Analyses

The BWAQ global score showed negative linear correlations with the age (r = −0.38; *p* < 0.0001) and years of education (r = −0.23; *p* < 0.0001).

Moreover, positive linear correlations were reported between the BWAQ global score and Impulsivity assessed by BIS-11 (r = 0.22; *p* < 0.05) and the BDI score (r = 0.30; *p* < 0.01), confirming an adequate convergent validity with the dimensions related to the addiction. 

## 4. Discussion

This study was aimed to develop and evaluate the psychometric properties of a scale to assess binge-watching behavior. To discriminate the pathological addictive behavior from the normal one was another aim of the study. The validity and reliability of the BWAQ were tested with both exploratory and confirmatory factor analyses in independent samples. EFA and CFA resulted in a four-factor model of the BWAQ with acceptable psychometric properties and fit. 

The four factors identified (i.e., Craving, Dependency, Anticipation, Avoidance) cover the range of the behavioral aspects involved in potential binge-watching addiction. Specifically, the Craving subscale allows detecting the pleasure experienced during watching, involving the mood dimension. This scale captures the strong urges and the intense desire to act. Moreover, it involves the interpersonal problems consequent to watching. Intense craving is a typical feature of addictive disorders and a key factor in the maintenance and relapse of behavioral addiction and substance one [44]. For this reason, highlighting the presence of craving in BW could help in delineating this possible “novel” behavioral addiction. 

The Dependency scale refers to the core characteristic of a pathological addiction behavior characterized by compulsivity (i.e., the implementation of repetitive acts perceived as not in line with one’s overall goal [45]) and loss of control. It covers household, sleep, and occupational-related problems. The items of the BWAQ included in this scale support the features that define it. The Anticipation scale and the Avoidance scale describe other aspects not usually analyzed in BW but potentially associated with problematic behavior, which can evolve in addiction. In particular, the first one refers to the anticipatory pleasure that promotes the implementation of the behavior over time. The second one refers to the tendency to minimize its impact in daily life, causing the individual to seek a justification for his/her behavior [46]. The analysis of all these aspects involved in addictive behaviors makes BWAQ very useful to understand the various facets of binge-watching. It allows us to understand better the personal aspects of BW other than purely motivational ones, as highlighted by other authors [26], with relevant clinical implications for further interventions.

These results are supported by the correlational analyses that indicated a higher tendency to engage BW associated with greater depression and impulsivity levels, confirming an adequate capacity of the questionnaire to assess some aspects associated with problematic or addictive behaviors [44].

Another aim of this study was to discriminate against pathological addictive behavior from the normal one. BW can be considered both highly entertaining and as an obsessive/compensatory behavior and could be placed in a continuum from normal to pathological. For these reasons, defining the border in which this behavior becoming maladaptive appears important since the possibility of pathologizing daily behavior is a serious risk that could influence clinical practice [47].

Some authors defined binge-watching as problematic when five consecutive episodes occur [28]. However, this definition is unclear and completely ignores that an equivalent amount of viewing time may cause problems for some individuals but not others [26]. Furthermore, it neglects the psychological implication behind this behavior. Therefore, we tried to focus on the negative impact of problematic binge-watching. We propose a cut-off score to support the hypothesis of a continuum from normal to pathological behavior. Considering our cut-off scores, BW is experienced as a problematic or addictive behavior by three participants out of one hundred. These results agree with other studies that found a similar percentage of behavioral addictions (e.g., eating, gambling, internet, sex, exercise) in the general population [48], both supporting the validity of the tool in identifying risk and proposing an addictive behavior spectrum also for the BW. Further studies would be distinguished where excessive BW is the consequence of mental disorders (e.g., depression, anxiety) or pathological distress. To determine possible comorbidity with other behavioral addictions also appears relevant.

TV series watching satisfies the need for entertainment, increasing well-being, and positive affect [1], and there are no interests in over pathologizing this widespread activity. However, problematic binge-watching could compromise multiple life areas (e.g., family, friendship, work) and physiological and psychological aspects before being identified (e.g., [2,21,22]). In this sense, more studies aimed at exploring BW and understanding several aspects associated with it are needed. We aimed to provide a rating scale capable of assessing this behavior before it becomes uncontrollable. A similar assessment, allowing an early evaluation of the risk of pathological BW, can allow a timely adoption of adequate strategies to counteract the pathological aspects of BW. An additional suggestion is offered by this study regarding the analysis of demographic variables. The results highlighted the presence of higher levels of BW in younger and people with lower education. These results are only preliminary; further investigations are needed, including deepening analyses on socio-demographical data.

## 5. Limitations

The present study clearly shows some limitations. Although the short version of the questionnaire, with 20 items, allows a rapid assessment, it reduces its capability to analyze other aspects involved in BW behavior and addiction (e.g., coping and emotional regulation, personality traits) and to deeply explore the dimensions reported (e.g., interpersonal relationship, the social impact of the behavior). Although the EFA indicated strong validity of the tool, some limits were highlighted by the CFA. It is known that the interpretation of CFA fit indices has limitations. However, the values that indicated a weakness in the model goodness would suggest implementing the instrument and verifying its stability over time. The sample’s characteristics represent another limit. The survey was mainly disseminated in the TV series fan communities to define the users’ main characteristics. This allows us to collect data in a sample characterized by Italian young adults and students; therefore, it does not cover all the population, reducing the generalizability of the results. Moreover, a limit is the adoption of a cut-off that considered the standard deviation from the mean. Further studies should be done on different populations or comparing different scales for the BW to verify whether these cut-offs are robust and how the BWAQ scores are distributed in different populations. Another limit could be the lack of comparison between the BWAQ and the other questionnaires adopted to measure the BW behavior. We are confident that further studies will overcome these limitations. Finally, this scale was validated in an Italian-speaking population, and further studies should test their validity in different cultures and languages.

## 6. Conclusions

Despite some limitations, our results emphasize that BWAQ has appropriate psychometric properties, and it constitutes a promising questionnaire for the emerging binge-watching research area. Another strength of BWAQ is the possibility of differentiating between people who adopt maladaptive watching behavior from those who use TV-series as leisure and entertainment activities. Therefore, this questionnaire may enable researchers to improve binge-watching research significantly.

Considering the increase of the BW in the general population, especially in younger people, and the declination of this behavior as a possible behavioral addiction, further research should be conducted on both problematic and unproblematic BW to define the link with other psychopathological aspects.

Potentially, understanding these relationships would have strong clinical implications in terms of prevention (i.e., defining the risk factors) and intervention (i.e., highlighting the intervention areas).

## Figures and Tables

**Table 1:** Characteristics of the samples for the EFA and CFA.

	Sample for EFA (N = 638)	Sample for CFA (N = 639)	Total Sample (N = 1277)
Age, mean (SD)	22.56 (5.81)	22.67 (6.07)	22.61 (5.94)
Females, N (%)	466 (73)	466 (73)	932 (73)
Years of education, mean (SD)	13.81 (2.99)	14.09 (3.43)	13.95 (3.22)
Educational status, N (%)			
Middle school	110 (17)	101 (16)	211 (17)
High school	372 (58)	347 (54)	719 (56)
Undergraduate	100 (16)	120 (19)	220 (17)
Graduate	42 (7)	59 (9)	101 (8)
Specialized	12 (2)	12 (2)	24 (2)
Occupational status, N (%)			
Student	464 (73)	477 (74)	941 (74)
Unemployed	28 (4)	31 (5)	59 (5)
Household	9 (1)	5 (1)	14 (1)
Physical occupation	55 (9)	50 (8)	105 (8)
Intellectual occupation	82 (13)	76 (12)	158 (12)
Marital status			
Single	389 (61)	411 (64)	800 (62)
Engaged	205 (32)	178 (28)	383 (30)
Married	40 (6)	47 (7)	87 (7)
Divorced	4 (1)	3 (1)	7 (1)

EFA: Explorative Factorial Analysis; CFA: Confirmative Factorial Analysis.

**Table 2:** Eigenvalues and Explained Variances of BWAQ factors.

	Initial Eigenvalues			Rotation Sum of Squared Loading		
Factor	Eigenvalues	Explained Variance (%)	Cumulative Explained Variance (%)	Eigenvalues	Explained Variance (%)	Cumulative Explained Variance (%)
1	9.06	41.17	41.17	4.94	22.44	22.44
2	1.67	7.60	48.77	3.07	13.96	36.40
3	1.38	6.29	55.06	3.04	13.82	50.21
4	1.10	5.01	60.07	2.17	9.85	60.07

**Table 3:** Loading of items in BWAQ after Oblimin rotation.

	Factor 1	Factor 2	Factor 3	Factor 4
Item_1		0.81		
Item_2		0.80		
Item_3	0.70			
Item_4			0.85	
Item_5				0.61
Item_6		0.50		0.45
Item_7			0.44	
Item_8				0.60
Item_9	0.65			
Item_10	0.56			
Item_11	0.73			
Item_12	0.63			
Item_13		0.74		
Item_14	0.52			
Item_15		0.73		
Item_17				0.72
Item_18	0.61			
Item_19	0.69			
Item_20	0.83			
Item_21				0.38
Item_22			0.76	
Item_23	0.77			

Only Factor Loading ≥ 0.35 are reported. For better clarity, the original number of each item is reported.

**Table 4:** The estimated four-factor model of the BWAQ.

	Standardized Factor Loading	Internal Consistency (Cronbach α)	Composite Reliability (CR)	Average Variance Extracted (AVE)
FACTOR 1: Craving		0.91	0.91	0.53
Item 9	0.76			
Item 10	0.72			
Item 11	0.82			
Item 12	0.64			
Item 14	0.61			
Item 18	0.66			
Item 19	0.75			
Item 20	0.80			
Item 23	0.73			
FACTOR 2: Dependency		0.82	0.82	0.53
Item 1	0.69			
Item 2	0.72			
Item13	0.69			
Item15	0.79			
FACTOR 3: Anticipation		0.75	0.75	0.50
Item 4	0.65			
Item 7	0.74			
Item 22	0.72			
FACTOR 4: Avoidance				
Item 5	0.71	0.81	0.82	0.53
Item 8	0.84			
Item 17	0.64			
Item 21	0.70			

For better clarity, the original number of each item is reported.

**Table 5:** BWAQ factors correlation matrix (*p* < 0.01).

	Factor 1	Factor 2	Factor 3
Factor 1	1.00		
Factor 2	0.73	1.00	
Factor 3	0.78	0.58	1.00
Factor 4	0.75	0.76	0.60

**Table 6:** Mean, Standard Deviation, and Cut-Offs of the BWAQ global score and subscales.

	Mean	Std. Dev.	Cut-Off
Global Score	33.42	17.64	Moderate: ≥51 and <69 Problematic: ≥69
Craving	16.31	10.29	Moderate: ≥27 and <37 Problematic: ≥37
Dependency	7.64	3.79	Moderate: ≥12 and <15 Problematic: ≥15
Anticipation	5.99	3.01	Moderate: ≥ 9 and <12 Problematic: ≥12
Avoidance	3.48	3.56	Moderate: ≥7 and <10 Problematic: ≥10

## Appendix A

**Table A1:** Binge-Watching Addiction Questionnaire. The English translation is shown in parentheses.

	Mai (Never) (0)	Raramente (Rarely) (1)	Qualche Volta (Sometimes) (2)	Spesso (Often) (3)	Sempre (Always) (4)
Quante volte sei rimasto/a a guardare serie TV più di quanto avresti voluto? (How many times have you been watching TV series more than you would have liked?)					
2. Ti capita di trascurare le faccende domestiche per passare più tempo a guardare serie TV? (Do you happen to neglect household chores to spend more time watching TV series?)					
3. Ti capita spesso di leggere recensioni e opinioni riguardo nuove serie televisive? (Do you often read reviews and opinions about new TV series?)					
4. Le persone che frequenti si lamentano per la quantità di tempo che passi a guardare serie TV? (Do people you hang out with complain about the amount of time you spend watching TV series?)					
5. Ti capita di controllare le nuove uscite on-demand prima di fare qualche altra cosa di importante? (Do you happen to check out the new on-demand releases before doing anything else important?)					
6. Cerchi di minimizzare quando qualcuno ti fa notare il tempo che tu trascorri a guardare serie TV? (Do you try to minimize when someone points out the time you spend watching TV series?)					
7. Quante volte ti trovi a distogliere l’attenzione da pensieri negativi con il pensiero consolatorio della tua serie TV preferita? (How many times do you find yourself diverting your attention from negative thoughts with the consoling thought of your favorite TV series?)					
8. Ti capita di pregustare il momento in cui guarderai nuovamente una serie TV? (Do you happen to foretaste the moment you will watch a TV series again?)					
9. Ti capita di pensare che la tua vita senza le serie TV sarebbe noiosa, vuota e senza gioia? (Do you happen to think that your life without the TV series would be boring, empty, and joyless?)					
10. Ti capita di scattare, alzare la voce o rispondere bruscamente se qualcuno ti disturba mentre guardi una serie TV? (Do you happen to react abruptly, raise your voice, or rudely reply if someone disturbs you while you are watching a TV series?)					
11. Dormi di meno per restare alzato/a fino a tardi per guardare una serie TV? (Do you sleep less to stay up late to watch a TV series?)					
12. Ti capita di concentrarti col pensiero sulle serie TV e fantasticare sull’evolversi della trama? (Do you happen to concentrate on your thoughts on TV series and fantasize about the evolution of the plot?)					
13. Ti capita di scoprirti a dire “ancora un’altra puntata e spengo” quando guardi una serie TV? (Do you happen to find yourself saying “one more episode and I’ll turn it off” when you watch a TV series?)					
14. Cerchi di minimizzare o di nascondere quanto tempo passi a guardare serie TV? (Do you try to minimize or hide how much time you spend watching TV series?)					
15. Ti capita spesso di sentiti depresso/a, irritabile, o nervoso/a quando non riesci a guardare una serie TV? (Do you often feel depressed, irritable, or nervous when you can’t watch a TV series?)					
16. Ti capita di scegliere di passare più tempo a guardare una serie TV anziché uscire con gli altri? (Do you happen to choose to spend more time watching a TV series rather than hanging out with others?)					
17. Ti capita di sentirti bene quando riesci nuovamente a guardare una serie TV? (Do you happen to feel good when you are able to watch a TV series again?)					
18. Ti capita di pensare che le persone sovrastimino il tempo che passi a guardare le serie TV? (Do you happen to think that people overestimate the time you spend watching TV series?)					
19. Ti interessi alle nuove serie TV? (Are you interested in new releases TV series?)					
20. Pensare ai momenti in cui guardi la tua serie TV preferita ti aiuta a gestire i tuoi momenti di stress? (Does thinking about the moments when you watch your favorite TV series help you manage your stressful moments?)					

For a better clarity the original number of each item is reported.
